# Mobilization with movement enhances early rehabilitation outcomes in knee osteoarthritis: a six-week randomized controlled trial

**DOI:** 10.1186/s12998-026-00636-0

**Published:** 2026-03-22

**Authors:** Shahnaz Hasan, Amir Iqbal, Abeer Ramadan Ibrahim, Reham H. Diab, Zaheen A. Iqbal, Tabinda Hasan, Shahnawaz Anwer, Ahmad H. Alghadir

**Affiliations:** 1 Department of Physical Therapy and Health Rehabilitation, College of Applied Medical Sciences, Majmaah University, Al-Majmaah, 11952 Saudi Arabia; 2 Rehabilitation Research Chair, Department of Rehabilitation Health Sciences, College of Applied Medical Sciences, King Saud University, P.O. Box. 10219, Riyadh, 11433 Saudi Arabia; 3 Medical Rehabilitation Sciences Department, Faculty of Applied Medical Sciences, Umm Al-Qura University, Mecca, Saudi Arabia; 4 Department of Physical Therapy, College of Medical Rehabilitation Science, Taibah University, Madina, Saudi Arabia; 5 Department of Physiotherapy, School of Allied Health Sciences, Manav Rachna International Institute and Studies (MRIIRS), Faridabad, India; 6 Department of Basic Sciences, College of Medicine, Princess Nourah Bint Abdulrahman University, P.O. Box. 84428, Riyadh, 11671 Saudi Arabia; 7 Department of Rehabilitation Sciences, The Hong Kong Polytechnic University, Hong Kong, China

**Keywords:** Knee osteoarthritis, Mobilization with movement, Manual therapy, Isometric training, Quadriceps strength, Rehabilitation

## Abstract

**Background:**

Exercise is a first-line approach for knee osteoarthritis (KOA), yet pain during movement may limit adherence. Mulligan’s mobilization with movement (MWM) is proposed to facilitate pain-free exercise and hasten early clinical gains.

**Objective:**

To determine whether adding MWM to isometric training (IT) improves pain, quadriceps strength, and disability over six weeks compared with IT alone.

**Methods:**

Two-arm, double-masked RCT in adults with radiographic KOA (Kellgren–Lawrence grade 2–3). Participants were randomized to MWM + IT or IT-only (*n* = 25/group), 5 supervised sessions/week for 6 weeks. Outcomes: pain (VAS), quadriceps strength (strain-gauge dynamometry), disability (WOMAC) measured at baseline, weeks 1, 3, and 6. Primary analyses used linear mixed-effects models with fixed effects for Group, Time, and Group × Time and a random intercept for Subject; joint Wald tests evaluated factor significance.

**Results:**

Significant Time effects indicated improvement across outcomes (VAS χ^2^(3) = 456.338, *p* < 0.0001; STN χ^2^(3) = 186.876, *p* < 0.0001; WOMAC χ^2^(3) = 1515.459, *p* < 0.0001). Group×Time interactions were significant for VAS (χ^2^(3) = 170.550, *p* < 0.0001), STN (χ^2^(3) = 204.327, *p* < 0.0001), and WOMAC (χ^2^(3) = 71.041, *p* < 0.0001), demonstrating faster and larger six-week gains with MWM + IT. The Group main effect was significant for VAS (χ^2^(1) = 42.599, *p* < 0.0001) and non-significant for STN (χ^2^(1) = 0.613, *p* = 0.4336) and WOMAC (χ^2^(1) = 0.019, *p* = 0.8901).

**Conclusion:**

Adding MWM to IT accelerated and amplified six-week improvements in pain, strength, and function compared with IT alone. Early integration of MWM may enhance pain-free exercise and short-term rehabilitation outcomes in KOA. Larger multicentre trials with extended follow-up and economic evaluation are warranted.

**Trial registration:**

ClinicalTrials.gov Identifier: NCT05577403; prospectively registered on 12 /07/2021 (last updated on 14/09/2025).; available at https://clinicaltrials.gov/study/NCT05577403.

## Introduction

Knee osteoarthritis (OA) is a prevalent degenerative joint disorder that contributes substantially to pain, functional disability, and reduced quality of life among middle-aged and older adults worldwide [1, 2]. It is characterized by progressive loss of articular cartilage, subchondral bone changes, and synovial inflammation, a leading cause of chronic pain mobility impairment, and health care burden [3]. Pain, stiffness, and muscle weakness, particularly of the quadriceps and periarticular muscles, are the key clinical features that limit daily function and increase the risk of falls [4, 5]. Conservative management is central to the treatment of knee OA, with physiotherapy playing a critical role in reducing pain, restoring mobility, and improving strength [6]. Exercise therapy, especially quadriceps strengthening, has been consistently recommended to mitigate functional decline [7]. Active exercises such as isotonic strengthening, closed kinetic chain training, and neuromuscular exercises can improve pain and function [8, 9].

In patients with symptomatic KOA, quadriceps weakness arises from both muscle atrophy and arthrogenic muscle inhibition (AMI). AMI, which is a reflex response triggered by joint pathology, inflammation, and altered afferent input [5, 10], impairs voluntary activation. It also reduces motor unit recruitment and decreases force, leading to persistent weakness and functional limitations. Dynamic isotonic exercises may be poorly tolerated early in rehabilitation. Movement-related nociceptive input can worsen inhibitory reflexes and reinforce maladaptive motor patterns. In contrast, isometric contractions recruit motor units without joint movement, reducing mechanical stress on sensitized structures [10, 11]. Emerging evidence suggests that isometric exercise can provide acute analgesic effects, modulate nociceptive input, and enhance neuromuscular activation [12, 13]. By reducing pain and partially restoring quadriceps activation, isometric contractions may help to overcome AMI and support progressive strengthening.

Isometric strengthening was performed first to target neural inhibition and minimize joint stress followed by the adjunct manual therapy, Mulligan’s Mobilization with Movement (MWM), was applied in early KOA rehabilitation. This sequential approach was designed to first optimize neuromuscular activation with isometric exercise, then assess the additive effect of subsequent MWM on pain modulation and motor relearning.

Among these, isometric exercises, which involve muscle contraction without joint movement, are widely used in knee OA rehabilitation due to their ability to strengthen periarticular muscles with minimal joint stress [11]. Quadriceps and hamstring isometrics are particularly effective in improving muscle activation and reducing pain sensitivity [12]. However, pain during active movement often limits patient adherence to therapeutic exercise, necessitating adjunctive manual therapy techniques to enhance outcomes [14].

Mulligan’s Mobilization with Movement (MWM) is a manual therapy technique that combines sustained accessory joint glides applied by the therapist with the patient’s simultaneous active physiological movement [15]. This approach aims to correct altered joint mechanics, reduce pain through neurophysiological mechanisms, and improve functional movement patterns [16]. Previous studies have shown that MWM provides immediate pain relief and enhance knee function in individuals with OA [17, 18]. Importantly, weight-bearing MWMs, such as medial glide and medial rotation techniques, may optimize proprioceptive input and neuromuscular control, thereby complementing strengthening interventions [19].

Although both isometric strengthening and MWM have demonstrated individual efficacy in knee OA management, limited randomized controlled trials have examined their combined effects. When combined with MWM, isometric exercises may enhance motor relearning, facilitate pain-free strengthening, and improve functional capacity. Therefore, this study aims to determine whether adding MWM to a supervised IT programme improves pain, quadriceps strength, and disability over six weeks compared with IT alone using linear mixed-effects modelling to analyse repeated measures. We hypothesized that the MWM + IT would produce greater improvement trajectories (Group X Time) in pain reduction, muscle strength, and functional outcomes compared to IT alone.

## Materials and methods

### Study design

This study was conducted using a double-masked, two-arm, parallel group, randomized controlled trial design.

### Study participants and setting

The study was conducted at the outpatient rehabilitation unit of the Physiotherapy and Health Rehabilitation Centre, Majmaah University, Saudi Arabia. Participants were recruited between November 2022 and December 2023 through referrals from rheumatologists and rehabilitation physicians. Eligibility screening was completed in three stages: (1) an initial questionnaire and telephone interview, (2) physical examination by a rheumatologist or rehabilitation physician, and (3) independent confirmation by the study assessor.

#### Inclusion and exclusion criteria

Participants were eligible for inclusion if they had a clinical diagnosis of OA confirmed by radiographic evidence corresponding to Kellgren–Lawrence grade 2 or 3, with involvement of the medial tibiofemoral compartment. Eligible individuals were between 40 and 65 years of age, with unilateral or bilateral knee OA; in cases of bilateral involvement, the more symptomatic knee was selected for the study. Additional criteria required a history of knee OA symptoms within the past year and pain localized to or around the knee joint.

Exclusion criteria included sever knee deformities such as varus or valgus malalignment, fixed flexion deformity, significant joint instability or significant deformities of the hip or spine, or if they had central or peripheral nervous system disorders. Additional exclusions were applied to those who had received intra-articular or steroid injections within the previous three months, undergone lower limb surgery in the past six months, or were unable to adhere to the treatment protocol or attend the required number of sessions.

### Ethical considerations

Ethial approval was obtained from the Research Ethics Committee, King Saud University (RRC-2021-17, dated 13/09/2021). Furthermore, the study protocol was prospectively registered online to “ClinicalTrials.gov (PRS) under assigned trial identifier: NCT05577403 on dated 12/07/2021. All procedures adhered to the Declaration of Helsinki, and written informed consent was obtained from all participants.

### Sample size estimation

An a priori power analysis was performed in G*Power for the group × time interaction in a two-arm repeated-measures design (4 measurements; test family: *F* tests → ANOVA, repeated measures—within–between interaction). Using the most conservative pilot outcome (WOMAC; pilot *n* = 12, 6 per group), the interaction effect was partial η^2^ = 0.261 (Cohen’s f = 0.594); the pilot also suggested a correlation among repeated measures of ~ 0.75. With α = 0.05, power (1–β) = 0.80, and a nonsphericity correction ε = 0.75, the required analysis sample is *N* = 38 (19 per group). Allowing 20–25% attrition, the recruitment target is *N* = 48–50 (24–25 per group) to ensure ≥ 38 completers for the primary analysis.

### Study procedure and sample randomization

**Fig. 1:**
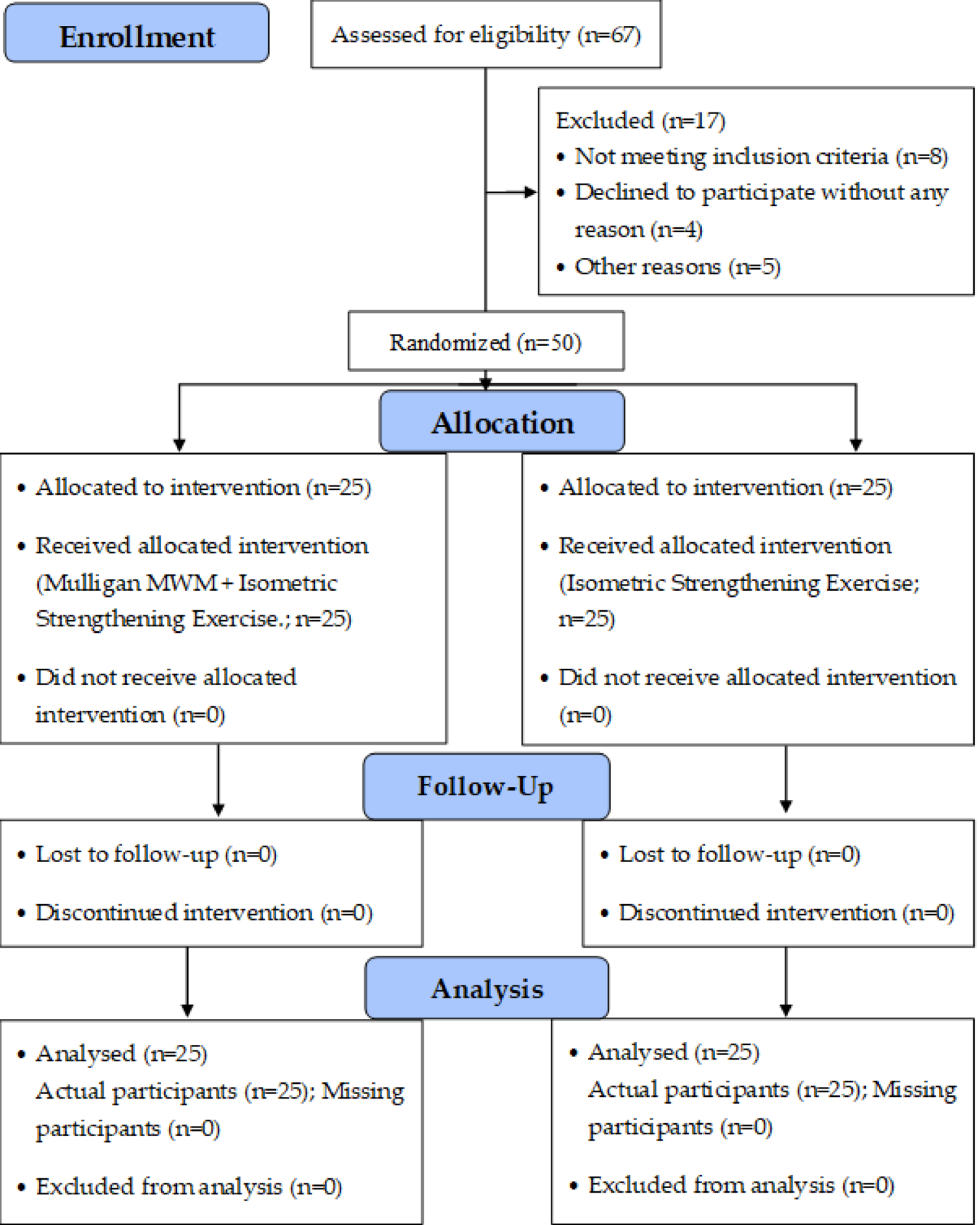
A CONSORT (2025) flow diagram depicts the study procedure, including enrollment, randomization, allocation, follow-up, and analysis

A total of 50 participants meeting the eligibility criteria were enrolled. After baseline assessments, participants were randomly allocated into two groups (MWM + IT Group and IT-Only Group, *n* = 25 each) using a computer-generated randomization sequence prepared by an independent researcher. Allocation concealment was maintained, and the outcome assessor (one assistant physiotherapist) was blinded to group assignment. Two senior physical therapists, each responsible for a specific intervention, delivered the treatments. One therapist administered Mulligan MWM, while the other provided isometric strengthening exercises. To ensure treatment fidelity and reduce bias, both therapists were blinded to the participants’ group allocations. For each outcome measure (VAS, STN, and WOMAC), three measurements were recorded at each time point (Baseline [Day 0], Week 1, Week 3, and Week 6). The average of these three values was used for statistical analysis. Although pain and function were also monitored during the first treatment week (Day 2, Day 4, and Day 6), these interim measurements were not included in the final analysis. Furthermore, A CONSORT (2025) flow diagram shows the study procedures, including the participants’ enrollment, eligibility assessement, randomization, allocation to intervention group, follow-up, and data analysis, as presented in Fig. 1.

### Study interventions

Interventions were administered over a six-week period, with five sessions per week in both groups. Participants in MWM + IT group received Mulligan’s MWM followed by isometric strengthening exercises, while IT-Only group’s participants performed the similar isometric strengthening exercise without MWM. All participants were advised to maintain their daily activities but were instructed to avoid receiving any additional physiotherapy or medical treatment during the trial period.

MWM was performed according to Mulligan’s guidelines [16], with the technique selected based on immediate pain relief during assessment. The therapist manually applied a sustained accessory tibiofemoral glide (medial glide or medial rotation, based on assessment) while the participant actively performed knee flexion–extension.

The glide was applied with moderate force to achieve pain-free movement and maintained throughout the active range. Intensity was adjusted individually to ensure complete symptom relief during movement, in accordance with Mulligan principles.

*Medial glide* Non-weight-bearing: Participant supine, while the therapist applied a medial tibiofemoral glide during active knee flexion and extension.

*Weight-bearing* Participant stood with the affected foot on a stool while the therapist maintained the medial glide during active knee flexion–extension.

*Medial rotation* The therapist applied a medial rotational force to the proximal tibia while the participant actively flexed and extended the knee.

*Progression strategy* If pain occurred in both weight-bearing and non-weight-bearing positions, treatment began in supine. Functional tasks were introduced sequentially as follows: Stepping the affected leg on/off a stool, stepping on a stool and moving the knee forward/backward, Stepping up and down a stool. Each task was performed for 2 sets of 10 repetitions, progressing only when the activity was pain-free. Most participants advanced to the third activity by the third or fourth session.

*Dosage and integration* Each treatment session included four sets of 10 repetitions of MWM distributed across non-weight-bearing and functional tasks with 60-second rest intervals between sets. Total MWM time was 10–15 min per session. To reinforce neuromotor adaptation, participants were asked to walk a few steps after each session to integrate the corrected movement pattern [19].

Isometric strength training: Following MWM (Group A) or as a stand-alone intervention (Group B), participants performed isometric strengthening exercises targeting the quadriceps, hamstrings, and gluteal muscles. Participants sat with their knee flexed to 60 degrees on the Isomove device and contracted their quadriceps isometrically with maximum force for 5 s, resting 10–15 s between repetitions and 1–2 min between sets. They performed three sets of 10 repetitions, five days a week, for six weeks. Each session lasted 20–25 min.

Isometric training is considered a safe and effective modality for improving muscle activation and function in knee OA [11, 13, 20].

Isometric quadriceps exercise: Supine with a towel roll under the knee, participants tightened their thigh muscles to straighten the knee for 5 s performing 3 sets of 10 repetitions, 5 days a week for 6 weeks.

Straight leg raise (SLR) exercise (Supine): While lying flat on their backs, participants performed a strong isometric quadriceps contraction before lifting their lower limbs 10 degrees, holding for 5 s. This exercise included three sets of 10 repetitions, five days a week, for six weeks.

Straight leg raise (SLR) exercise (Prone): Participants lie prone with legs straight and head resting on their arms, tightened their glutes and hamstrings, raised the leg as high as possible toward the ceiling, and held for 5 s then lowered the leg, rested for 2 s, and repeated. This exercise included three sets of 10 repetitions, five days a week, for six weeks.

### Outcome measures

The following validated tools were used for assessment:

- Pain intensity was measured using the Visual Analogue Scale (VAS), a reliable and tool for musculoskeletal pain assessment. The VAS has demonstrated high test–retest reliability (ICC > 0.90), strong construct validity, and responsiveness in osteoarthritis populations [21]. Participants’ current pain levels were rated on a 0–10 scale, with 10 indicating the maximum pain and 0 indicating no pain.
- Isometric strength (IS) test: Quadriceps muscle strength (STN) was evaluated using the ISOMOVE Quadriceps maximum isometric peak torque on the dominant side. Measurement was done with an ISOMOVE dynamometer (ISO-MANSW-IT; Tecnobody, Dalmine (BG), Italy), software version 0.0.1. Isokinetic dynamometers, which measure muscle performance during movement at a constant speed, provide reliable and valid measures of velocity, joint position, and torque [22]. The ISOMOVE’s reliability for measuring quadriceps strength has been validated [23, 24]. Reported values for isometric knee extensor peak torque at 60° knee flexion include ICC (intraclass correlation coefficient) = 0.088 (0.73–0.94), CV (coefficient of variation) = 7.19, and SEM (standard error of measurement) = 9.98 [24]. Before testing, participants were familiarized with the apparatus. Participants seated with backs supported, hips at 90°, and knees at 60° flexion [25], straps secured the pelvis, mid-thighs, and chest to prevent movement, while the shin pad was positioned 5.1 cm (2 inches) above the medial malleolus (the bony prominence on the inner ankle). Participants then crossed their arms over their chests and performed three 5-second maximal contractions, with 2-minute rests between trials. The average peak torque from these contractions was used as the baseline measurement.
- Disability and functional status were assessed using the Western Ontario and McMaster Universities Osteoarthritis Index (WOMAC), a disease-specific tool widely applied in OA clinical trials. The WOMAC includes 24 items divided into three domains: Pain (5 items), Stiffness (2 items), and Physical Function (17 items). In this study, the Likert version (0–4 scale per item) was used. Total WOMAC scores ranged from 0 to 96, with higher scores indicating greater pain and disability. The WOMAC demonstrates excellent internal consistency (Cronbach’s α > 0.80), test–retest reliability (ICC > 0.85), and responsiveness in clinical trials of knee OA [13, 26].

### Statistical analysis

All data were analysed using SPSS (version 21, IBM Corp., Armonk, NY, USA), adhered to a complete-case approach (no attrition). Descriptive statistics were computed for demographic variables, and normality of distribution was assessed using the Shapiro–Wilk test. Primary analyses employed linear mixed-effects models (LMMs) for each outcome with fixed effects for Group (MWM + IT vs. IT-only), Time (Baseline, Week 1, Week 3, Week 5), and Group × Time interaction, plus a random intercept for Subject to account for repeated measures. Maximum likelihood estimation was used. Joint Wald tests (χ^2^) evaluated Time, Group, and Group × Time; two-sided *p* < 0.05 denoted statistical significance. Effect sizes for Group and Group × Time were quantified as ηp^2^ from a Type III ANOVA with subject fixed effects for interpretability. The coefficients, SEs, z-statistics, and *p*-values were reported. All analyses adhered to a complete-case approach (no attrition). Significance was set at *p* < 0.05.

## Results

### Participant characteristics

A total of 67 individuals were assessed for eligibility; 17 were excluded (8 did not meet inclusion criteria, 4 declined to participate, and 5 for other reasons). Fifty participants were randomized: 25 were allocated to MWM + IT and 25 to IT-Only. No participants were lost to follow-up or discontinued the intervention. All randomized participants were included in the analysis (25 per group). Demographic characteristics were comparable between groups (Table 1): age, height, weight, and BMI showed no statistically significant between-group differences. Sex distribution was uniform (all female). Baseline outcomes were balanced for quadriceps strength and WOMAC disability, whereas baseline pain (VAS) was higher in the MWM + IT group than the IT-only group (mean ± SD: 7.78 ± 0.88 vs. 6.10 ± 0.99; *p* < 0.0001). This baseline VAS imbalance is acknowledged in interpretation of the Group main effect and underscores the emphasis on the Group × Time interaction for pain.

**Table 1:** Demographics and baseline outcomes’ details (*n* = 25/group)

Variables	MWM + IT group (*n* = 25)	IT-only group (*n* = 25)	*P*-value
Age	55.36 ± 6.29	55.12 ± 7.25	0.901
Sex (n, %)	F: 25 (100%)	F: 25 (100%)	1.000
Weight	68.60 ± 6.76	65.80 ± 4.64	0.094
Height	155.92 ± 4.10	155.92 ± 4.10	0.821
BMI	28.20 ± 2.39	27.14 ± 1.32	0.059
VAS0	7.78 ± 0.88	6.10 ± 0.99	0.001*
STN0	9.09 ± 1.58	9.44 ± 1.65	0.456
WOMAC0	24.64 ± 3.30	24.52 ± 4.15	0.910

*Significance value, if *p* < 0.05; BMI: Body Mass Index; 0: Baseline Scores; VAS: Visual Analogue Scale; STN: Muscle Strength; WOMAC: Western Ontario and McMaster Universities Osteoarthritis Index; MWM: Mulligan’s Mobilization with Movement; IT: Isometric Training

Table 2 presents Shapiro–Wilk normality results for each outcome by group and time. Most distributions were approximately normal; notable exceptions occurred for VAS in the IT-only group at baseline and week 1, with occasional departures at week 6. Given the cell sizes and the use of linear mixed-effects models—generally robust to mild non-normality—these deviations are unlikely to bias the primary inferences.

**Table 2:** Summarising the normality test of normal distribution, using a Shapiro–Wilk normality by group × time (exact *p*-values) (*N* = 25/group)

Variables (*n* = 25)	Groups (*n* = 25/group)	Shapiro-Wilk Test of Normality (95% CI; 2-tailed)		
		Statistics	*p*-value	Normal distribution
VAS0	MWM + IT	0.955	0.3274	Yes
	IT-Only	0.862	0.003*	No
STN0	MWM + IT	0.932	0.0964	Yes
	IT-Only	0.831	0.0008*	No
WOMAC0	MWM + IT	0.944	0.1798	Yes
	IT-Only	0.926	0.0699	Yes

*Significance value, if *p* < 0.05; BMI: Body Mass Index; 0: Baseline Scores; VAS: Visual Analogue Scale; STN: Muscle Strength; WOMAC: Western Ontario and McMaster Universities Osteoarthritis Index; MWM: Mulligan’s Mobilization with Movement; IT: Isometric Training

### Linear mixed-effects

#### Group × time interaction (across variables)

Table 3 demonstrates large interaction effects across outcomes, indicating steeper improvement trajectories in the MWM + IT arm over six weeks: VAS ηp^2^=0.532, STN ηp^2^=0.577, and WOMAC ηp^2^=0.321 (all *p* < 0.0001 by joint tests). These magnitudes indicate that adding MWM explains a substantial share of variance in change over time—consistent with greater and faster pain reduction, strength gains, and disability improvement relative to IT-only.

**Table 3:** Group × Time effect (ηp^2^ from Type III ANOVA with subject fixed effects) (*N* = 50)

Outcomes	Effect	F-value	df	*p*-value	ηp^2^
VAS	Group × time	54.576	3	0.001*	0.532
STN	Group × time	65.385	3	0.001*	0.577
WOMAC	Group × time	22.733	3	0.001*	0.321

*-Significant value, if *p* < 0/05; VAS: Visual Analogue Scale; STN: Muscle Strength; WOMAC: Western Ontario and McMaster Universities Osteoarthritis Index; ηp^2^: partial eta-squared (Effect sizes) from Type III fixed-effects ANOVA with subject fixed effects; used to provide interpretable magnitudes alongside mixed-model tests; († (ηp^2^): ~0.01 small, ~ 0.06 medium, ~ 0.14 large

#### Group main effect (across variables)

Table 4 shows a small overall between-group difference for pain (VAS ηp^2^≈0.091, *p* ≈ 0.001) and negligible offsets for strength (STN ηp^2^≈0.001, *p* ≈ 0.840) and disability (WOMAC ηp^2^≈0.003, *p* ≈ 0.491). Taken with Table 3, these results indicate that the benefit of adding MWM is expressed primarily as different trajectories over time rather than as a constant group gap across all time points.

**Table 4:** Group main effect (ηp^2^ from Type III ANOVA with subject fixed effects) (*N* = 50)

Outcomes	Effect	F-value	df	*p*-value	ηp^2^
VAS	Group (Overall)	14.452	1	0.001*	0.091
STN	Group (Overall)	0.041	1	0.840	0.001
WOMAC	Group (Overall)	0.477	1	0.491	0.003

*Significant value, if *p* < 0/05; VAS: Visual Analogue Scale; STN: Muscle Strength; WOMAC: Western Ontario and McMaster Universities Osteoarthritis Index; ηp^2^: partial eta-squared (Effect sizes) from Type III fixed-effects ANOVA with subject fixed effects; used to provide interpretable magnitudes alongside mixed-model tests; († (ηp^2^): ~0.01 small, ~ 0.06 medium, ~ 0.14 large

#### Time main effect (across variables)

Table 5 confirms substantial within-subject improvement across the programme for all variables: VAS F(3) = 146.029, *p* < 0.0001, ηp^2^=0.753; STN F(3) = 59.801, *p* < 0.0001, ηp^2^=0.555; WOMAC F(3) = 484.947, *p* < 0.0001, ηp^2^=0.910. These large effects show marked reductions in pain and disability and meaningful strength gains over six weeks, consistent with effective supervised rehabilitation.

**Table 5:** Time main effect (ηp^2^ from Type III ANOVA with subject fixed effects) (*N* = 50)

Outcomes	Effect	F-value	df	*p*-value	ηp^2^
VAS	Time (Overall)	146.029	3	0.0001*	0.753†
STN	Time (Overall)	59.801	3	0.0001*	0.555†
WOMAC	Time (Overall)	484.947	3	0.0001*	0.910†

*-Significant value, if *p* < 0/05; VAS: Visual Analogue Scale; STN: Muscle Strength; WOMAC: Western Ontario and McMaster Universities Osteoarthritis Index; ηp^2^: partial eta-squared (Effect sizes) from Type III fixed-effects ANOVA with subject fixed effects; used to provide interpretable magnitudes alongside mixed-model tests; († (ηp^2^): ~0.01 small, ~ 0.06 medium, ~ 0.14 large

Within the MWM group, all participants commenced treatment in the non–weight-bearing position. The majority progressed to weight-bearing activities by the second session, and most advanced to functional stepping tasks by the third or fourth session. A small proportion required prolonged treatment at earlier stages due to persistent symptoms. Collectively, Time effects confirm that the supervised programme produced clinically important improvements across outcomes, while the Group × Time results (Table 3) clarify that those gains were greater and faster with MWM + IT.

### Adverse events

No adverse events were recorded.

## Discussion

The present randomized controlled trial examined the effects of Mulligan’s MWM combined with isometric exercise versus isometric exercise alone in patients with knee OA. Both interventions produced significant improvements in pain, quadriceps strength, and functional disability over six weeks, confirming the central role of exercise-based rehabilitation in knee OA management [8, 9]. Although both groups improved, participants who received MWM in addition to IT demonstrated earlier and more consistent within-group improvements, particularly in pain and functional disability by week 2.

Findings of this study align with previous reports that MWM provides immediate pain relief, enhances joint proprioception, and facilitates pain-free engagement in exercise [19, 27–29]. The proposed mechanisms include correction of positional faults within the tibiofemoral joint, stimulation of type II mechanoreceptors, reduction in nociceptive input, and improvements in motor control [13]. By alleviating pain during active movement, MWM may accelerate motor relearning and optimize the strengthening response. Quadriceps weakness is a well-established risk factor for the onset and progression of knee OA and contributes significantly to functional limitations [30]. In this trial, both groups improved quadriceps strength, supporting prior evidence that isometric strengthening enhances muscle activation, reduces pain, and improves functional capacity [22, 23]. Notably, Anwer and Alghadir (2014) demonstrated that a five-week isometric quadriceps program significantly improved quadriceps strength, pain, and WOMAC scores in knee OA patients compared to controls [23]. Our findings extend this evidence, suggesting that combining MWM with isometric training may accelerate functional recovery, even though between-group differences did not reach statistical significance.

The lack of significant between-group differences in our study may be due to limited sample size, the short intervention duration, or the relatively high efficacy of isometric exercises alone. Similar findings were reported by Abbott et al. [14], who found that both exercise and manual therapy improved outcomes in lower-limb OA, but differences between combined and stand-alone approaches were not always statistically significant. However, the within-group progression patterns observed in the MWM group, particularly earlier pain and function improvements, support the notion that MWM may provide adjunctive clinical benefit. Our results are also consistent with Kiran et al. [18], who showed that incorporating MWM into conventional rehabilitation produced superior functional outcomes compared to exercise alone in KOA. Taken together with the work of Anwer and colleagues, the current findings strengthen the argument that integrating manual therapy with strengthening exercises may enhance patient-centred outcomes in KOA rehabilitation.

### Clinical implications

The findings highlight that Mulligan’s MWM can accelerate early improvements in pain and functional disability when combined with isometric training, even though both interventions converge to similar outcomes by six weeks. For clinicians, this suggests that incorporating MWM in the initial phase of rehabilitation may help patients achieve faster symptom relief, improve adherence to strengthening programs, and enhance motivation during the often-challenging early treatment period. However, given the lack of long-term superiority, MWM should be viewed as a complementary strategy rather than a replacement for exercise therapy. Careful integration of MWM into individualized treatment plans may optimize short-term outcomes while maintaining focus on sustained functional improvement through ongoing exercise.

### Limitations and future directions

Several limitations must be acknowledged. First, the modest sample size (*n* = 50) may have limited statistical power to detect between-group differences despite observable clinical trends. Second, the trial duration was restricted to six weeks, and long-term sustainability of the improvements remains unknown. Third, the study included only patients with Kellgren–Lawrence grade 2–3 OA, limiting generalizability to those with early or advanced disease. Fourth, while participants were instructed to avoid additional physiotherapy or pharmacological treatments, adherence outside the clinic could not be strictly controlled. Finally, outcome measures were limited to VAS, STN, and WOMAC without incorporating biomechanical or imaging-based markers that could have provided mechanistic insights.

Future studies should aim to recruit larger, multicentre cohorts to improve generalizability and statistical power. Long-term follow-up is needed to determine whether the benefits of combined MWM and isometric training are sustained and whether they influence structural disease progression. Incorporating objective biomechanical assessments such as gait analysis, proprioceptive testing, or electromyography may clarify the mechanisms underlying observed improvements. Comparative effectiveness trials against other physiotherapy interventions, such as Maitland mobilizations, neuromuscular training, or resistance-based closed kinetic chain exercises, would help determine the unique contribution of MWM. Finally, inclusion of quality-of-life measures and cost-effectiveness analyses would further support clinical decision-making. Additionally, cost-effectiveness analyses are warranted to assess the feasibility of implementing MWM in routine practice. Such work will provide stronger evidence for guiding clinical decision-making and integrating MWM into standardized care pathways for knee osteoarthritis.

## Conclusion

Over six weeks, MWM + IT produced larger and faster improvements in pain, quadriceps strength, and disability than IT alone in KOA. These data support integrating MWM as an adjunct to exercise during the early rehabilitation phase to enhance symptom relief and functional gains. Longer-term and economic evaluations are needed to establish durability and value. Future large-scale, long-term trials are needed to establish the durability, underlying mechanisms, and cost-effectiveness of integrating MWM into standard exercise therapy for knee OA.

## Data Availability

The dataset that drew the result conclusion has been presented in the study and will be available from the corresponding author upon a reasonable request.
